# Resting metabolic rate in obese diabetic and obese non-diabetic subjects and its relation to glycaemic control

**DOI:** 10.1186/1756-0500-6-382

**Published:** 2013-09-26

**Authors:** Azza O Alawad, Tarig H Merghani, Mansour A Ballal

**Affiliations:** 1 Department of Physiology, Faculty of Medicine, University of Al-Neelain, Khartoum, Sudan; 2 Department of Physiology, Faculty of Medicine, University of Tabuk, Tabuk, Saudi Arabia; 3 Department of Physiology, Faculty of Medicine, University of Science and Technology, Khartoum, Sudan

**Keywords:** Diabetes Mellitus, Glycated haemoglobin, Indirect calorimetry, Obesity, Oxygen consumption, Resting metabolic rate

## Abstract

**Background:**

Both obesity and type II diabetes mellitus are associated with insulin resistance and abnormal metabolic reactions. This study was conducted to evaluate resting metabolic rate in obese diabetic patients and to assess its relation to glycaemic control.

**Results:**

This is a case control study conducted in Gabir AbuEliz centre in Khartoum, Sudan. A random sample of 40 obese diabetic patients (cases) and 40 obese non-diabetic subjects (controls) were interviewed and examined clinically to exclude presence of acute or chronic medical illness. Haemoglobin A1c was measured for each participant using the “NycoCard Haemoglobin A1c test” (Axis -Shield/ Norway). Fasting blood sugar was measured using one touch(R) glucometer (LifeScan Canada Ltd). The PowerLab 8/35 with a gas analyzer (AD Instruments, Castle Hill Australia) was used for measurement of VO2, VCO2 and Respiratory exchange ratio (RER). Resting metabolic rate was calculated using the Weir equation. VO2 (mean+/-SD) ml/min was significantly higher among cases (209.9+/-42.7) compared to the controls (192.4+/-28.1), (P = 0.034). Similarly, VCO2 (mean+/-SD) ml/min was higher among cases (191.4+/-35.0) than controls (178.3+/-22.5), (P = 0.05). Resting metabolic rate “RMR” (mean+/-SD) kcal/day was higher in obese diabetic patients (1480.7 +/- 274.2) than obese non-diabetic subjects (1362.4+/- 184.8), (P = 0.027). Participants with high glycated haemoglobin had higher RMR than those with normal glycated haemoglobin (P = 0.016).

**Conclusion:**

It is concluded that resting metabolic rate is significantly higher in obese diabetic patients compared to obese non-diabetics, especially in those with poor glycaemic control.

## Background

Measurement of resting energy expenditure is essential for nutritional assessment, weight loss planning and optimum medical care. Indirect calorimetry is the primary method for metabolic rate measurement. It involves measurement of oxygen consumption (VO2) and carbon dioxide production (VCO2) for calculation of caloric burning rate. The relation between these gases and metabolic rate is defined by the Weir equation which is widely used for metabolic rate calculations [1,2]. Obesity, which is one of the most prevalent serious health problems worldwide, is associated with insulin resistance and type 2 diabetes mellitus. Insulin resistance in obese diabetic individuals is associated with abnormal metabolic reactions in skeletal muscle, liver and adipose tissue. Management and prevention of long term complications in diabetic patients depend on tight glycaemic control. Laboratory indicators of glycaemic control in a diabetic patient are haemoglobin A1c (Hb A1c) and fasting blood sugar (FBS). The quantity of Hb A1c within a red blood cell reflects the average level of glucose to which the cell has been exposed during its life-cycle. Measuring Hb A1c assesses the level of glycaemic control over the previous four weeks to three months. Normal value is about 5% of total haemoglobin in adults. It was recommended that a threshold of ≥6.5% HbA1c could be used to diagnose diabetes [3]. Previous studies reported higher resting metabolic rate among diabetic patients compared to the non-diabetics. Weyer and his colleagues in 1999 found progressive increase in resting metabolic rate in patients in whom their glucose tolerance deteriorated from normal glucose tolerance to impaired glucose tolerance to diabetes [4]. They attributed the rise in resting metabolic rate to metabolic changes that occur during early stages of diabetic development [4]. Fontvieille and other researchers suggested that the increased 24-h sedentary energy expenditure may play a role in the weight loss that is often observed in Type 2 diabetic subjects in addition to the energy loss from glycosuria [5]. Similar findings have been reported recently in severely obese diabetic patients [6]. The researchers suggested a new predictive equation for calculation of resting metabolic rate using gender, age, height, weight and diabetes mellitus as variables [6]. In an earlier study the race had been suggested as a variable as well [7]. The inconsistent results and the different variables suggested in these studies indicate that estimation of resting metabolic rate in obese diabetic patients using predictive equations may be misleading and requires further investigations, especially in the tropics. Results of a survey published in the past showed that basal metabolic rate values of people in the tropics appear to be lower than values estimated with WHO predictive equations [8]. On the other hand; there is paucity of data regarding relation of resting energy expenditure to glycaemic control in obese diabetic patients. This study was conducted to evaluate resting metabolic rate among these patients and to assess its relation to laboratory indicators of glycaemic control.

## Methods

This is a case control study conducted in Jabir Abu-Eliz centre in Khartoum- Sudan, in the period from January to August 2012. A random sample of 40 obese diabetic patients (cases) was selected from 190 patients attended for follow up during that period. A sample of 40 obese non-diabetic subjects (controls) was selected from the co-patients. Inclusion criteria for both groups were age 35 to 50 years old, body mass index ≥ 30 kg/m^2^, non-smoker, normotensive, afebrile and with no symptoms or signs of acute or chronic infection. Cases were known diabetics for at least one year, either on diet control (27 patients) or stopped taking their anti-diabetic medications for at least two days before presentation (13 patients), whereas controls were non-diabetics. Both groups were matched according to age, sex, height, weight, physical activity, socioeconomic status and area of residence. An anonymous interviewer-based questionnaire, requesting information about daily exercise and present health status was applied to each participant. Clinical examination was carried out to exclude signs of acute or chronic medical abnormalities. Height and weight of each participant was measured using standardized height and weight scales. Body mass index (BMI) was calculated as weight (in kilograms)/height (in meters^2^). Five micro litre of blood was taken for Haemoglobin A1c measurement using the “NycoCard Haemoglobin A1c test” (Axis -Shield/ Norway); with a measuring range of 4-15% HbA1c. Fasting blood sugar was measured using one touch® glucometer (LifeScan Canada Ltd). The PowerLab 8/35 (AD Instruments, Castle Hill Australia) data acquisition systems, comprising hardware and software, were used with bio amplifier, thermistor, spirometer, gas analyzer, gas mains chamber and a computer windows 7 program to record and analyze physiological signals from participants. They allowed measurement of oxygen consumption (VO2), carbon dioxide production (VCO2), respiratory ventilation (VE) and respiratory exchange ratio (RER). All participants were fasting overnight. They were allowed to rest in a thermally neutral room for 30 minutes before measurements. Each test took less than 30 minutes, with at least 5 minutes steady state. The steady state was defined as minimal variation (< 10%) in VO2, VCO2, VE and RER. Resting metabolic rate was calculated using the Weir equation as follows [1,2]: RMR (kcal) = [(VO2) (3.941) + (VCO2)(1.11)] 1440 min.

The research conforms to the ethical principles of medical research developed by the World Medical Association Declaration of Helsinki [9]. Ethical clearance was given by the Research Committee (Faculty of Medicine/ University of Al-Neelain). Approval was obtained from the Ministry of Health. Written consents were obtained from each participant before entry into the study. All data obtained with questionnaire and biochemical analysis were analyzed using the Statistical Package for the Social Sciences (SPSS) version 19. The chi square test was used to test distribution of categorical variables. The differences between test and control groups were assessed with the student’s t test. Statistical significance was accepted when P value is ≤ 0.05.

## Results

Table 1 describes characteristics of cases (n = 40) and controls (n = 40) in the study group. Male: Female ratio was 1:1.5 in both groups. Statistical analysis showed insignificant difference between the two groups in age (P = 0.564), height (P = 0.094), weight (P = 0.486), body mass index (P = 0.328) and surface area (P = 0.078). Table 2 shows that VO2 (mean ± SD) ml/min was significantly higher among cases (209.9 ± 42.7) compared to the control group (192.4 ± 28.1), (P = 0.034). Similarly, VCO2 (mean ± SD) ml/min was higher among cases (191.4 ± 35.0) than controls (178.3 ± 22.5), (P = 0.05); whereas the mean respiratory exchange ratio (RER) is almost equal in the two groups (0.93 ± 0.16 and 0.93 ± 0.11 respectively). Table 3 shows higher resting metabolic rate “RMR” (mean ± SD) among cases (1480.7 ± 274.2; CI = 1393.0-1568.4) than controls (1362.4 ± 184.8; CI = 1303.3-1421.5), (P = 0.027). RMR was significantly higher among participants with abnormal glycated haemoglobin than those with normal glycated haemoglobin (P = 0.016; Table 4). Insignificant difference was found between those with normal and those with abnormal fasting blood sugar (P = 0.561; Table 5).

**Table 1:** Characteristics of cases and controls in the study group

**Parameter**	**Study group**	**Min**	**Max**	**Mean**	**SD**	**P value**
Age (y)	Cases	35	50	45.03	5.09	0.564
	Controls	35	50	44.33	5.69	
Height (m)	Cases	1.49	1.81	1.63	0.08	0.094
	Controls	1.55	1.83	1.66	0.08	
Weight (kg)	Cases	69	105	85.00	7.70	0.486
	Controls	78	105	86.52	11.35	
BMI (kg/m^2^)	Cases	30.02	40.37	32.04	2.08	0.328
	Controls	30.04	39.85	32.29	2.06	
Surface area (m^2^)	Cases	1.69	2.30	1.96	0.128	0.078
	Controls	1.85	2.31	2.01	0.126	

**Table 2:** Oxygen consumption (VO2), carbon dioxide production (VCO2) and respiratory exchange ratio (RER) among diabetic and non-diabetic participants

	**VO2**		**VCO2**		**RER**	
**Parameter**	**Diabetic**	**Non-diabetic**	**Diabetic**	**Non-diabetic**	**Diabetic**	**Non-diabetic**
	**N = 40**	**N = 40**	**N = 40**	**N = 40**	**N = 40**	**N = 40**
Minimum	153.0	143.0	155.0	129.0	0.7	0.8
Maximum	311.0	261.0	309.0	228.0	1.2	1.1
Mean	209.9	192.4	191.4	178.3	0.93	0.93
SD	42.7	28.1	35.0	22.5	0.16	0.11
CI	196.2-223.5	183.4-201.4	180.2-202.6	171.1-185.5	0.88-0.98	0.90-0.97
P value	0.034		0.050		0.868	

**Table 3:** Resting metabolic rate (RMR among diabetic and non-diabetic participants

	**RMR/day**	
**Parameter**	**Diabetic**	**Non-diabetic**
	**n = 40**	**n = 40**
Minimum	1155.6	1057.3
Maximum	2238.8	1787.3
Mean ± SD	1480.7 ± 274.2	1362.4 ± 184.8
CI	1393.0-1568.4	1303.3-1421.5
P value	0.027	

**Table 4:** Resting metabolic rate (RMR) in relation to hemoglobin A1c

	**RMR/day**	
**Parameter**	**HbA1c <6.5%**	**HbA1c ≥6.5%**
	**n = 46**	**n = 34**
Minimum	1057.3	1155.6
Maximum	1898.5	2238.8
Mean ± SD	1363.4 ± 195.4	1500.1 ± 273.1
CI	1305.4-1421.5	1404.9-1595.4
P value	0.016	

**Table 5:** Resting metabolic rate (RMR) in relation to fasting blood sugar

	**Fasting blood sugar**	
**Parameter**	**(< 126 mg/dL)**	**(≥ 126 mg/dL)**
	**n = 50**	**n = 30**
Minimum	954.7	1050.6
Maximum	2824.6	2926.4
Mean ± SD	1358.6 ± 330.8	1407 ± 403.5
CI	1264.5-1452.6	1256.4-1557.8
P value	0.561	

## Discussion

Measurement of energy expenditure is an important element in the estimation of energy requirements in man. In addition to its importance in scientific research, it is becoming an essential tool in maintenance of a healthy body weight. Obese subjects, especially those with type II diabetes mellitus, may need special consideration because insulin resistance in these individuals is associated with abnormal metabolic reactions. In this study, a significant difference in RMR was found between obese diabetic and obese non-diabetic subjects. The difference was associated with higher rate of oxygen consumption and carbon dioxide production that caused rise in resting metabolic rate among the diabetic patients. In previous studies, many mechanisms have been suggested to explain similar findings. One of the proposed mechanisms was an increase in gluconeogenesis [10]. It has been suggested that the increased free fatty acid concentration in diabetic patients contributes to the excessive rates of gluconeogenesis and therefore to increased rate energy expenditure in these patients. Indian patients with type II diabetes mellitus showed higher rate of energy expenditure correlating with increased endogenous release of glucose from the liver [5]. In support with this mechanism, a reduction in resting energy expenditure was reported, following improvement in glycaemic control [11]. Similarly in this study, we found lower resting metabolic rates in patients with well controlled diabetes mellitus, as indicated with normal glycated haemoglobin, compared to those with poor control. However, insignificant relation with fasting blood sugar was also found. This indicates that, glycaemic control has long term rather than short term effect on resting energy expenditure. Other proposed mechanisms include abnormal protein metabolism [12], increased sympathetic activity [13] and hyperglucagonaemia [14]. Diabetic patients in poor control show an increased protein turnover, requiring an increased protein synthesis, which is associated with higher rate of energy release [12,15]. There is an evidence that sympathetic nervous system activity is increased in hyperinsulinemic individuals with type 2 diabetes compared with non-diabetic control subjects [13]. On the other hand, it has been found that lowering glucagon levels during insulin deprivation decreased the resting metabolic rate by more than 7% [14]. These findings indicate that high levels of glucagon, which is a known finding in diabetes mellitus, may be responsible for the rise in energy expenditure among diabetic patients [15]. Recently it has been suggested that glucagon might be responsible for both the hyperglycaemia and the occasional hypoglycaemia seen in diabetic patients [16].

It is worth noting that, variation in resting metabolic rate between individuals of the same ethnicity is not uncommon. In a previous study, it was reported that basal metabolic rate values in a population varied from as low as 1027 kcal/day to as high as 2499 kcal; with a mean of 1500 kcal/day [17]. Similarly in our study, resting metabolic rate varied within diabetic patients from 1155.6 to 2238.8 kcal/day with a mean of 1480.7 kcal/day, and within the non-diabetic subjects from 1057.3 to 1787.3 kcal/day with a mean of 1362.4 kcal/day. The inter-individual differences in RMR within each group may be explained by variation in age, sex, muscle bulk and level of circulating hormones. It is worth noting that, variation in rate of energy release within the same subject “intra-individual variation” may contribute to this finding. In one study, intra-individual variation was found to be within a range of 5% [18].

One of the major limitations in this study is matching cases and controls. Although our objective measurements showed insignificant difference between the two groups in weight and height, we can’t exclude recall bias in other factors like age, residence and physical activity. In addition, we can’t confirm that one third of our diabetic participants missed their anti-diabetic medications two days before the study. However, this appears to be a common practice [19]. In one study, more than 50% of patients with insulin dependent diabetes mellitus missed their insulin dose [19].

## Conclusion

This study showed that resting metabolic rate is significantly higher among obese diabetic patients compared to obese non-diabetics. High resting metabolic rate has a significant relation with abnormal level of glycated haemoglobin but not with high fasting blood sugar, indicating that poor glycaemic control has a long term, rather than a short term effect on resting metabolic rate.

## Competing interests

The authors declare that they have no competing interests.

## Authors’ contributions

AO conceived the study, carried out the laboratory work and helped to draft the manuscript. TH participated in data collection, performed the statistical analysis and drafted the article. MA participated in study design, supervised the study and helped to draft the manuscript. All authors read and approved the final manuscript.

## References

[B1] de Weir JB New methods for calculating metabolic rate with special reference to protein metabolism J Physiol 1949 109 1 9 15394301 10.1113/jphysiol.1949.sp004363 PMC1392602

[B2] Mansell PI Macdonald IA Reappraisal of the Weir equation for calculation of metabolic rate AJP - Regu Physiol 1990 258 6 R1347 R1354 10.1152/ajpregu.1990.258.6.R1347 2360685

[B3] American Diabetes Association Diagnosis and classification of diabetes mellitus Diabetes Care 2010 33 Suppl 1 S62 S69 20042775 10.2337/dc10-S062 PMC2797383

[B4] Weyer C Bogardus C Pratley RE Metabolic factors contributing to increased resting metabolic rate and decreased insulin-induced thermogenesis during the development of type 2 diabetes Diabetes 1999 48 1607 1614 10.2337/diabetes.48.8.1607 10426380

[B5] Fontvieille AM Lillioja S Ferraro RT Schulz LO Rising R Ravussin E Twenty-four-hour energy expenditure in Pima Indians with type 2 (non-insulindependent) diabetes mellitus Diabetologia 1992 35 753 759 1511802 10.1007/BF00429096

[B6] Huang KC Kormas N Steinbeck K Loughnan G Caterson ID Resting metabolic rate in severely obese diabetic and nondiabetic subjects Obesity research 2004 53 11 1395 1398 10.1038/oby.2004.101 15166305

[B7] Martin K Wallace P Rust PF Gravey WT Estimation of resting energy expenditure considering effects of race and diabetes status Diabetes Care 2004 27 1405 1411 10.2337/diacare.27.6.1405 15161796

[B8] Henry CJK Rees DG New predictive equations for the estimation of basal metabolic rate in tropical people Eur J Clin Nutr 1991 45 177 185 1824042

[B9] World Medical Association Medical Ethics Committee Updating the WMA declaration of Helsinki Wld Med J 1999 45 11 13

[B10] Felig P Wahren J Hendler R Influence of maturity-onset diabetes on splanchnic glucose balance after oral glucose ingestion Diabetes 1978 27 121 126 10.2337/diab.27.2.121 624441

[B11] Franssila-Kallunki A Groop L Factors associated with basal metabolic rate in patients with type 2 (non-insulin-dependent) diabetes mellitus Diabetologia 1992 35 962 966 10.1007/BF00401426 1451954

[B12] Gougeon R Pencharz PB Marliss EB Effect of NIDDM on the kinetics of whole-body protein metabolism Diabetes 1994 43 318 328 10.2337/diab.43.2.318 8288057

[B13] Huggett RJ Scott EM Gilbey SG Stoker JB Mackintosh AF Mary DA Impact of type 2 diabetes mellitus on sympathetic neural mechanisms in hypertension Circulation 2003 108 3097 3101 10.1161/01.CIR.0000103123.66264.FE 14676139

[B14] Charlton MR Nair KS Role of hyperglucagonemia in catabolism associated with type 1 diabetes: effects on leucine metabolism and the resting metabolic rate Diabetes 1998 47 11 1748 1756 10.2337/diabetes.47.11.1748 9792544

[B15] Nair S Halliday D Garrow J Increased energy expenditure in poorly controlled type 1 insulin-dependent diabetic patients Diabetologia 1984 27 13 16 6147290 10.1007/BF00253494

[B16] Cryer PE Minireview: Glucagon in the pathogenesis of hypoglycemia and hyperglycemia in diabetes Endocrinology 2012 153 3 1039 1048 10.1210/en.2011-1499 22166985 PMC3281526

[B17] Johnstone AM Murison SD Duncan JS Rance KA Speakman JR Koh YO Factors influencing variation in basal metabolic rate include fat-free mass, fat mass, age, and circulating thyroxine but not sex, circulating leptin, or triiodothyronine Am J Clin Nutr 2005 82 5 941 948 16280423 10.1093/ajcn/82.5.941

[B18] Adriaens MP Schoffelen PF Westerterp KR Intra-individual variation of basal metabolic rate and the influence of daily habitual physical activity before testing Br J Nutr 2003 90 2 419 423 10.1079/BJN2003895 12908903

[B19] Elbagir MN Eltom MA Rosling H Berne C Glycaemic control of insulin-dependent diabetes mellitus in Sudan: influence of insulin shortage Diabetes Res Clin Pract 1995 30 1 43 52 10.1016/0168-8227(95)01145-5 8745205

